# Differences in antioxidant activities of outdoor- and indoor-cultivated *Agaricus brasiliensis*, and protective effects against carbon tetrachloride-induced acute hepatic injury in mice

**DOI:** 10.1186/1472-6882-14-454

**Published:** 2014-11-24

**Authors:** Daisuke Yamanaka, Masuro Motoi, Akitomo Motoi, Naohito Ohno

**Affiliations:** Laboratory for Immunopharmacology of Microbial Products, School of Pharmacy, Tokyo University of Pharmacy and Life Sciences, 1432-1 Horinouchi, Hachioji, Tokyo, 192-0392 Japan; Toei Shinyaku Co., Ltd, 1-11-23 Shimorenjyaku, Mitaka, Tokyo, 181-0013 Japan

**Keywords:** *Agaricus blazei*, Cultivation condition, Sunlight, Hepatitis, Antioxidative activity

## Abstract

**Background:**

*Agaricus brasiliensis* (*A. brasiliensis*) is a medicinal mushroom that exerts various pharmacological actions. We previously demonstrated that different cultivation conditions altered the activity of the polyphenol-related enzymes from this mushroom. However, the influence of cultivation conditions on the antioxidant activity of the fruiting bodies remains unclear. Therefore, in this study we compared the antioxidative effects of fruiting bodies of *A. brasiliensis* cultivated outdoors and indoors. In addition, we assessed whether different cultivation methods affected the hepatoprotective effects against CCl_4_-induced liver injury.

**Methods:**

We assessed the antioxidative effects of mushrooms cultivated in open-air or indoors using the DPPH radical-scavenging assay. Furthermore, we prepared experimental feeds containing outdoor- or indoor-cultivated *A. brasiliensis*. Acute liver injury was induced by CCl_4_ injection in mice that consumed feed containing outdoor- or indoor-cultivated *A. brasiliensis*. The hepatoprotective effects of these mushrooms were then evaluated by monitoring the reduction in the circulating levels of alanine aminotransferase, aspartate aminotransferase, and lactate dehydrogenase. The significance of the differences between the means was assessed using Student’s *t*-test. Finally, histopathological analysis of liver was performed.

**Results:**

In the DPPH assay, the antioxidant activity of outdoor-cultivated *A. brasiliensis* was higher than that of indoor-cultivated mushroom. Moreover, in the mouse model of CCl_4_-induced hepatitis, the oral administration of outdoor-cultivated *A. brasiliensis* reduced liver damage significantly, but indoor-cultivated mushrooms failed to inhibit hepatitis. The hepatoprotective effects of outdoor-cultivated *A. brasiliensis* were observed even when ingestion commenced only 1 day before CCl_4_ injection, and these effects were not affected by excessive heat treatment.

**Conclusions:**

Outdoor cultivation significantly enhanced the antioxidative activity of *A. brasiliensis* fruiting bodies. In addition, outdoor-cultivated *A. brasiliensis* was more effective at protecting against CCl_4_-induced liver injury in mice than mushrooms grown in a greenhouse.

## Background

*Agaricus brasiliensis* (*A. brasiliensis*) S. Wasser *et al.* (also known as *A. blazei* Murrill *sensu Heinemann*, *A. rufbtegulis* and *A. subrufescens*) [1–3] is a medicinal mushroom used worldwide as a biological response modifier [4, 5]. To date, many reports demonstrated beneficial effects of *A. brasiliensis* on human health. For example, natural killer cell activity was upregulated after the intake of the fruiting body of *A. brasiliensis* without any adverse effects [6]. The immunoenhancing effects of this mushroom are principally exerted by abundant β-glucans [7, 8], and several innate immune receptors such as Toll-like receptor (TLR) 2 [9], TLR4 [10], and dectin-1 [11] also contribute to the augmentation of host immune systems stimulated by *A. brasiliensis*. Evidence of pharmaceutical activity has improved the reliability of *A. brasiliensis* as a functional food.

In addition to immunoenhancing activities, we identified additional beneficial effects of *A. brasiliensis*, such as the protection against concanavalin A- or LPS-induced murine liver injury, after oral administration in water extracts [6]. The oral administration of extracts of *A. brasiliensis* fruiting bodies or mycelium also exhibited hepatoprotective effects in a model of CCl_4_-induced hepatitis [12, 13]. Although the underlying mechanism(s) behind its hepatoprotective effects remain unclear, the antioxidative activity of *A. brasiliensis* could contribute to its activity because inhibiting the generation of free radicals is important for protecting against CCl_4_-induced hepatic injury [13, 14]. Therefore, the antioxidative properties of this mushroom may be important for the treatment of free radical-induced hepatitis.

Hepatitis remains an incurable disease. In particular, viral hepatitis is highly infectious, and so is a global health issue. Many patients who are unable receive effective pharmaceutical agents choose to use traditional medicines such as *A. brasiliensis* due to its hepatoprotective activities. Today, many commercial products based on this mushroom are available, and patients can select one of these products. However, we previously demonstrated that different *A. brasiliensis* cultivation conditions (such as open air or indoors) affected its protein and chemical components significantly. In particular, outdoor cultivation strongly upregulated the activity of peroxidase and laccase [15]. These enzymes contribute to the degradation or composition of phenolic compounds, and, thus, cultivation conditions may influence their antioxidative properties and related biological activities. Therefore, to assess the optimum use of *A. brasiliensis*, we investigated the effect of cultivation conditions on its antioxidative activity.

In the present study, we assessed the influence of cultivation conditions on the antioxidant activities of three kinds of *A. brasiliensis* mushroom and compared the hepatoprotective effects of outdoor- and indoor-cultivated *A. brasiliensis* on CCl_4_-induced murine hepatic injury.

## Methods

### Animals and materials

Male ICR mice (4 weeks of age) were purchased from Japan SLC (Shizuoka, Japan). The mice were housed in a specific pathogen-free environment, and were used at 5–6 weeks of age. All experiments were performed in accordance with the guidelines for laboratory animal experiments provided by the Tokyo University of Pharmacy and Life Sciences, and the Committee for Laboratory Animal Experiments at Tokyo University of Pharmacy and Life Sciences (P13-47) approved each experimental protocol. 1,1-diphenyl-2-picrylhydrazyl (DPPH) free radical was purchased from Tokyo Chemical Industry Co., Ltd, Tokyo, Japan. 6-Hydroxy-2,5,7,8-tetramethylchroman-2-carboxylic acid (Trolox) was purchased from Calbiochem Inc., CA, USA. Carbon tetrachloride was purchased from Wako Pure Chemical Co. (Osaka, Japan), and olive oil was obtained from Nikko Pharmaceutical Co., Ltd. (Gifu, Japan).

### *A. brasiliensis*fruiting bodies

*A. brasiliensis* strain KA21, deposited at National Institute of Technology and Evaluation, Japan with deposition number: FERM P-17695 was cultivated outdoors with sunlight or indoors avoiding sunshine, using the same compost. The fruiting bodies were harvested in Brazil, washed, and dried using hot air at 60°C or lower (Toei Shinyaku Co., Ltd., Tokyo, Japan). The Japanese commercial product of dried fruiting bodies from another strain of *A. brasiliensis*, cultivated indoors in Japan, was also purchased and tested as another indoor-cultivated sample. Outdoor- and indoor-cultivated *A. brasiliensis* KA21, and the indoor-cultivated Japanese sample were named KAOD, KAID, and JAID, respectively. The ITS-5.8S rDNA sequence of KAOD and JAID was analyzed using ITS4 and ITS5 primers at Techno-Suruga Laboratory Co., Ltd. (Shizuoka, Japan). KAOD and JAID showed 99.6% and 100% homology with *A. subrufescens* (GenBank accession number AY818654.1 and KJ541796.1, respectively).

### Measurement of ingredients

Japan Food Research Laboratories (Shibuya, Tokyo) measured the ingredients of *A. brasiliensis* fruiting bodies using the standard protocols recommended by the Resources Council, the Science and Technology Agency of Japan.

### Antioxidant assay

The ability of *A. brasiliensis* to scavenge DPPH free radicals was assessed using the previously described decolorization method, with minor modifications [16, 17]. Briefly, powdered mushrooms were extracted with 50% methanol (50 mg/mL) at 60°C for 60 min. Trolox dissolved in 50% methanol was used as a standard antioxidant. Diluted extracts (20 μL) were combined with 200 μL of 150 μmol/mL DPPH in 50% methanol. All samples were prepared in triplicate. Following incubation in the dark at room temperature for 30 min, the absorbance at 517 nm was read using a Safire microplate reader (Tecan, Salzburg, Austria). The effect of natural color changes of the samples was controlled for by using a blank, and the data was expressed as equivalents of Trolox.

### Feed preparation

Various doses of powdered fruiting bodies from *A. brasiliensis* were mixed into the basic synthetic diet AIN-93G (Oriental Yeast Co., Ltd, Tokyo, Japan), and cornstarch was substituted for *A. brasiliensis*. The doses of KAOD tested were 1, 3, and 10%, whereas KAID and JAID were tested at 3 and 10%, respectively. AIN-93G supplemented with cornstarch was used as the control diet. All feeds were admixed by Oriental Yeast Co., Ltd, and the molding and drying processes were carried out at 60°C or lower. Each test diet was irradiated with 30 kGy γ-rays for sterilization. The ingredients in the solid feed are shown in Table 1. To evaluate the heat stability of KAOD, AIN-93G and KAOD-10% were autoclaved and air-dried at 50°C, and named heat-treated AIN-93G and heat-treated KAOD, respectively.

**Table 1:** **Ingredients in solid feed**

Ingredient	Content (g/kg diet)
Corn starch	397.486 - *R*
Casein	200.000
α-Corn starch	132.000
Sucrose	100.000
Soybean oil	70.000
Cellulose powder	50.000
AIN-93G Mineral Mix	35.000
AIN-93G Vitamin Mix	10.000
L-Cystine	3.000
Choline bitartrate	2.500
tert-butylhydroquinone	0.014
KAOD, KAID, or JAID	*R*

Control diet (AIN-93G): *R* =0.

KAOD-1%: *R* =10.

KAOD-3%: *R* =30.

KAOD-10%: *R* =100.

KAID-3%: *R* =30.

JAID-10%: R =100.

### Experimental protocol

**Figure 1:**
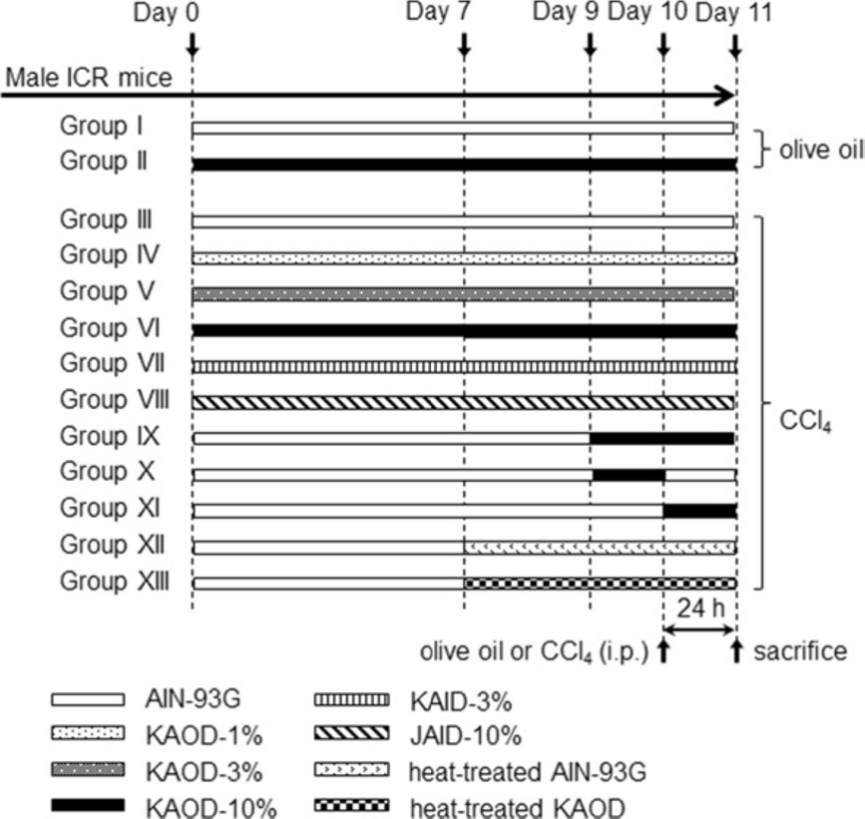
**Experimental protocol.**

For the chemical hepatic injury experiments, mice were divided randomly into 13 groups. All groups of mice were fed an experimental diet for 11 days. On the 10th day, hepatotoxicity was induced by intraperitoneal (i.p.) injection of CCl_4_ (4 ml/kg body weight of 1% CCl_4_ solution in olive oil). In the control group, mice received an i.p. injection of olive oil alone. The feeding design is shown in Figure 1. Mice in groups I and II received AIN-93G and KAOD-10%, respectively, during the experiment, and were treated with olive oil. Mice in groups III–VI received AIN-93G, KAOD-10%, KAOD-3%, and KAOD-1%, respectively. Mice in Groups VII and VIII received KAID-3% and JAID-10%, respectively. Groups IX were fed AIN-93G, but then received KAOD-10% on day 9 until the end of the experiment. Mice in Groups X or XI were fed AIN-93G, and then received KAOD-10% for 24 h before or after the injection of CCl_4_, respectively. Mice in Groups XII or XIII received AIN-93G, followed by heat-treated AIN-93G or heat-treated KAOD, respectively, from day 7 until the end of the experiment. All mice in Groups III– XIII were treated with CCl_4_ on day 10. Twenty-four hours after the i.p. administration of CCl_4_ or olive oil, mice were sacrificed by CO_2_ inhalation. Blood samples were collected by heart puncture, and the liver was removed and used to analyze biochemical markers of early acute hepatic damage and for histopathological evaluation.

### Measurement of serum ALT, AST, and LDH activities

Collected whole blood was allowed to stand at room temperature for 90 min, and then centrifuged (12,000 rpm for 10 min at 4°C) to obtain serum. The enzyme activities of alanine aminotransferase (ALT) and aspartate aminotransferase (AST) in serum were then measured using a Wako Transaminase CII-Test kit (Wako Pure Chemical Co.) following the manufacturer’s instructions. The color development was stopped using citric acid solution, and the optical density at 550 nm was measured using a microplate reader (MTP450; Corona Electric, Ibaraki, Japan). Lactate dehydrogenase (LDH) activity was assayed according to the instructions supplied with the commercial assay kit (NescoVL LD, Alfresa Pharma Co., Osaka, Japan). The absorbance at 340 nm was read using a Safire microplate reader (Tecan).

### Histopathological analysis

Livers were fixed in 10% formalin neutral buffered solution (pH 7.4) (Wako Pure Chemical Co.), embedded in paraffin blocks, and thinly sectioned. Tissue sections were stained with hematoxylin and eosin (HE). The Biopathology Institute Co., Ltd. (Oita, Japan) prepared paraffin blocks and carried out HE staining. The sections were then observed under a normal light microscope (Biozero BZ-8100 microscope, Keyence, Osaka, Japan).

### Statistical analysis

The significance of the differences between the means was assessed by Student’s *t*-test.

## Results

### Influence of cultivation conditions of the antioxidative activities of *A. brasiliensis*

**Figure 2:**
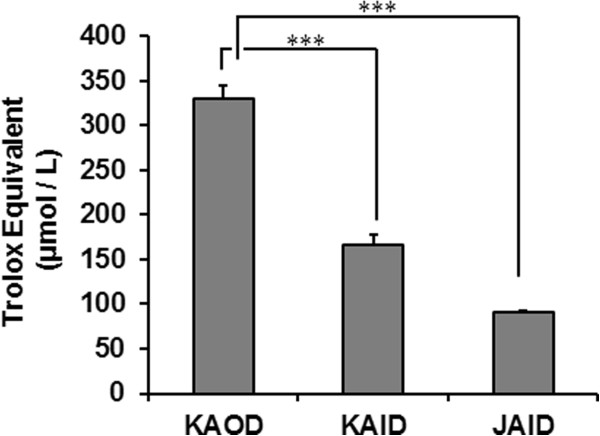
**Free radical scavenging activities of various** ***A. brasiliensis*** **.** The antioxidant capacities of KAOD, KAID, and JAID extracts (powder 50 mg/mL, supernatant) were measured using the DPPH radical-decolorization assay. Each sample was diluted and reacted with the DPPH reagent. Results are presented as mean (*n* =3) ± SD. Significant difference from KAOD extract: ***p <0.001.

We demonstrated previously that the enzymatic activities of *A. brasiliensis* fruiting bodies can be modulated by cultivation conditions, such as open-air or indoor growth [15]. Therefore, we first compared the ingredients in the fruiting bodies of *A. brasiliensis* and studied the antioxidant activities of KAOD, KAID, and JAID. As shown in Table 2, the basic composition of KAOD and JAID was similar (except for vitamin D and calcium), and there were no major differences between KAOD and KAID. Nevertheless, in the DPPH radical-scavenging assay, the antioxidant activity of KAOD extract was higher than that of KAID or JAID (Figure 2). These data suggest that outdoor cultivation augments the antioxidant capacity of *A. brasiliensis* fruiting bodies.

**Table 2:** **Basic nutrient composition comparison of cultivation methods**

	KAOD	KAID	JAID	Unit
Moisture	7.5	5.5	5.9	g
Energy	179	184	183	kcal
Protein	39.8	43.3	41.6	g
Fat	2.9	3.7	3.1	g
Carbohydrate	24.2	21.5	20.2	g
Fiber	18.9	19	22.7	g
Sodium	3.4	7.5	9.7	mg
Vitamin A (total caronene)	-	-	-	
Vitamin B1 (Thiamin)	0.79	1.41	0.52	mg
Vitamin B2 (Riboflavin)	3.5	4.01	5.49	mg
Vitamin B6	0.49	0.87	0.76	mg
Vitamin B12	0.27	-	-	μg
Niacin	48.8	47.7	53.4	mg
Pantothenic acid	20.2	23.9	15.9	mg
Folic acid	0.18	0.36	0.21	mg
Biotin	150	174	128	μg
Total vitamin C (Total c acid)	-	-	-	
Vitamin D	30.7	-	0.9	μg
Vitamin E (Total tocopherol)	-	-	-	mg
Vitamin K1 (Phylloquinone)	-	-	-	μg
Calcium	42.5	36.6	1.6	mg
Iron	11	11.8	7.76	mg
Phosphorus	994	987	1110	mg
Magnesium	98.9	105	114	mg
Potassium	2.84	2.96	2.67	g
Copper	16.5	12.3	1.65	mg
Iodine	-	-	-	
Manganese	0.67	0.7	0.82	mg
Selenium	51	120	24	μg
Zinc	10.9	19.7	16.4	mg
Total chromium	0.05	-	-	mg
Molybdenum	-	-	-	

-; not detected.

All data are shown for 100 g dry weight, and were measured by Japan Food Research laboratories.

### Effect of outdoor cultivated-*A. brasiliensis*on CCl_4_-induced hepatic injury

**Figure 3:**
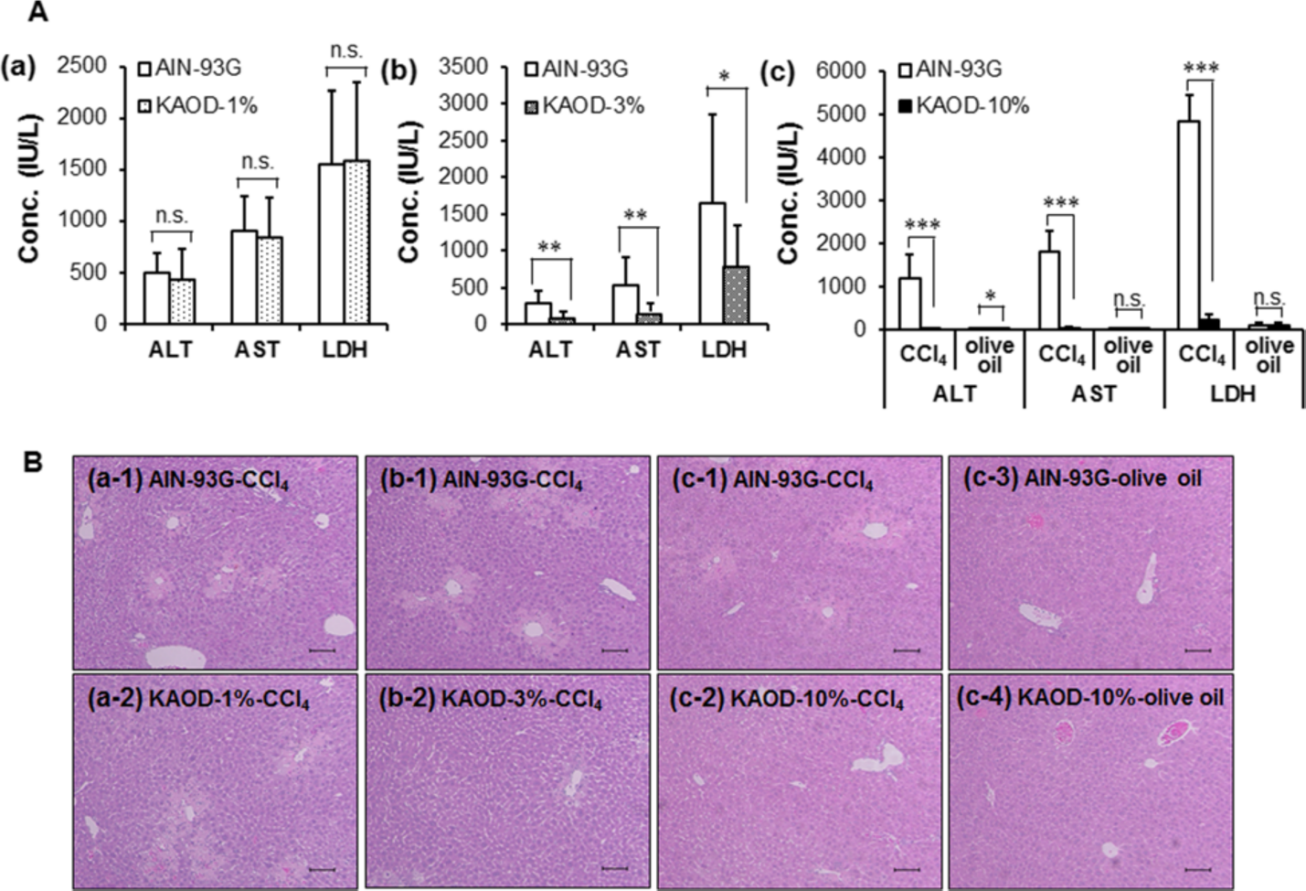
**Hepatoprotective effects of KAOD.** Serum and livers were isolated from ICR mice fed KAOD-1%, KAOD-3%, or KAOD-10% *ad libitum* for 11 days. **(A)** ALT, AST, and LDH activities in the serum from mice fed (a) KAOD-1% and injected with CCl_4_, (b) KAOD-3% and injected with CCl_4_ or (c) KAOD-10% and injected with CCl_4_ or olive oil were measured using commercially available kits. Data are presented as mean ± standard deviation, (a) *n* =6, (b) *n* =12, (c) *n* =7 (CCl_4_), *n* =3 (olive oil). Significant difference from AIN-93G: **p <0.01; ***p <0.001; n.s.: not significant. **(B)** HE-stained liver sections from mice administered with (a-1) AIN-93G with CCl_4_ (control for KAOD-1%), (a-2) KAOD-1% with CCl_4_, (b-1) AIN-93G with CCl_4_ (control for KAOD-3%), (b-2) KAOD-3% with CCl_4_, (c-1) AIN-93G with CCl_4_ (control for KAOD-10%), (c-2) KAOD-10% with CCl_4_, (c-3) AIN-93G with olive oil, or (c-4) KAOD-10% with olive oil were observed under a normal light microscope. Scale bar =100 μm.

Because KAOD yielded increased antioxidative activity, we next focused on its pharmacological activity. To evaluate whether the oral administration of *A. brasiliensis* whole fruiting bodies exerted hepatoprotective properties, we carried out an *in vivo* experiment using a mouse model of CCl_4_-induced hepatitis. As shown in Figure 3A, although KAOD-1% did not suppress the blood release of liver enzymes, 10 days of administration of KAOD-10% or KAOD-3% significantly inhibited CCl_4_-induced changes in the circulating levels of enzymes including ALT, AST, and LDH. In particular, mice fed KAOD-10% showed comparable levels of biochemical markers as olive oil-injected mice. Data obtained from histopathological studies supported the results of the biochemical analyses. (Figure 3B). Liver sections from control and KAOD-alone treated mice showed normal liver architecture. Injection with CCl_4_-induced obvious liver injury, which presented as large areas of extensive necrosis and a loss of hepatic architecture. KAOD prevented the development of CCl_4_-induced histological changes in a dose-dependent manner.

### The effect of cultivation conditions of the hepatoprotective activities of *A. brasiliensis*

**Figure 4:**
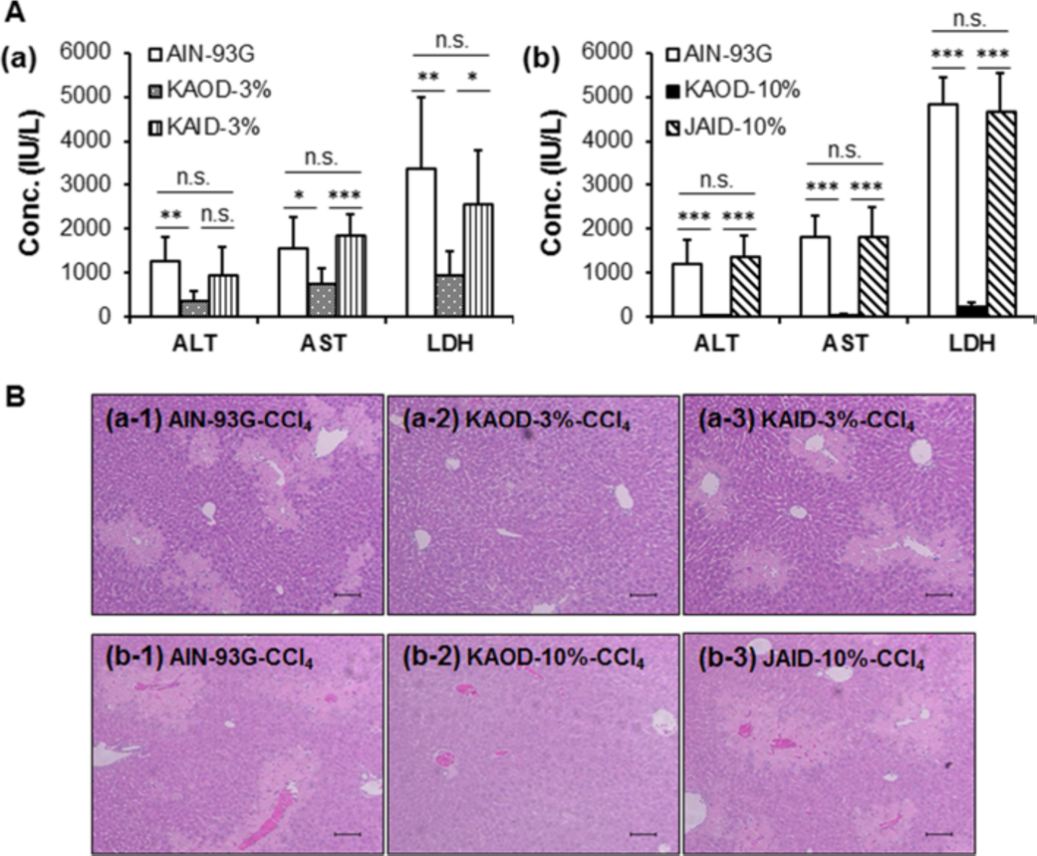
**Effect of cultivation conditions of the hepatoprotective activities of** ***A. brasiliensis*** **.** Serum and livers were isolated from ICR mice fed AIN-93G, KAOD-3%, KAOD-10%, KAID-3%, or JAID-10% *ad libitum* for 11 days. **(A)** ALT, AST, and LDH activities in the sera from (a) KAID-3% or KAOD-3% administered mice, or (b) KAOD-10% or JAID-10% administered mice were measured using commercially available kits. Data are presented as mean ± standard deviation, (a) *n* =6, (b) *n* =7. Significant difference from AIN-93G or (a) between KAID-3% and KAOD-3% or (b) JAID-10% or KAOD-10%: *p <0.05; **p <0.01; ***p <0.001; n.s.: not significant. **(B)** HE stained liver sections from mice administered (a-1) AIN-93G with CCl_4_ (control for KAOD-3% and KAID-3%), (a-2) KAOD-3% with CCl_4_, (a-3) KAID-3% with CCl_4_, (b-1) AIN-93G with CCl_4_ (control for KAOD-10% and JAID-10%), (b-2) KAOD-10% with CCl_4_, (b-3) JAID-10% with CCl_4_,. Scale bar =100 μm.

The above experiments defined the optimal dose of KAOD for hepatoprotection, therefore, we next prepared experimental diets containing KAID and compared its effects on outdoor- and indoor-cultivated *A. brasiliensis* KA21. Although KAOD-3% exerted hepatoprotective effects, KAID-3% did not inhibit the release of hepatic enzymes or prevent histopathological changes in the liver (Figure 4).

To confirm these effects, we prepared a commercially available product from another strain of *A. brasiliensis* cultivated indoors in Japan, and mixed it into the experimental diet as JAID-10%. Oral administration of JAID-10% did not suppress the acute liver injury induced by injection of CCl_4_ (Figure 4). These results strongly suggest that indoor cultivation significantly reduces the hepatoprotective activity of *A. brasiliensis* fruiting bodies.

### Optimization of the hepatoprotective properties of *A. brasiliensis*

**Figure 5:**
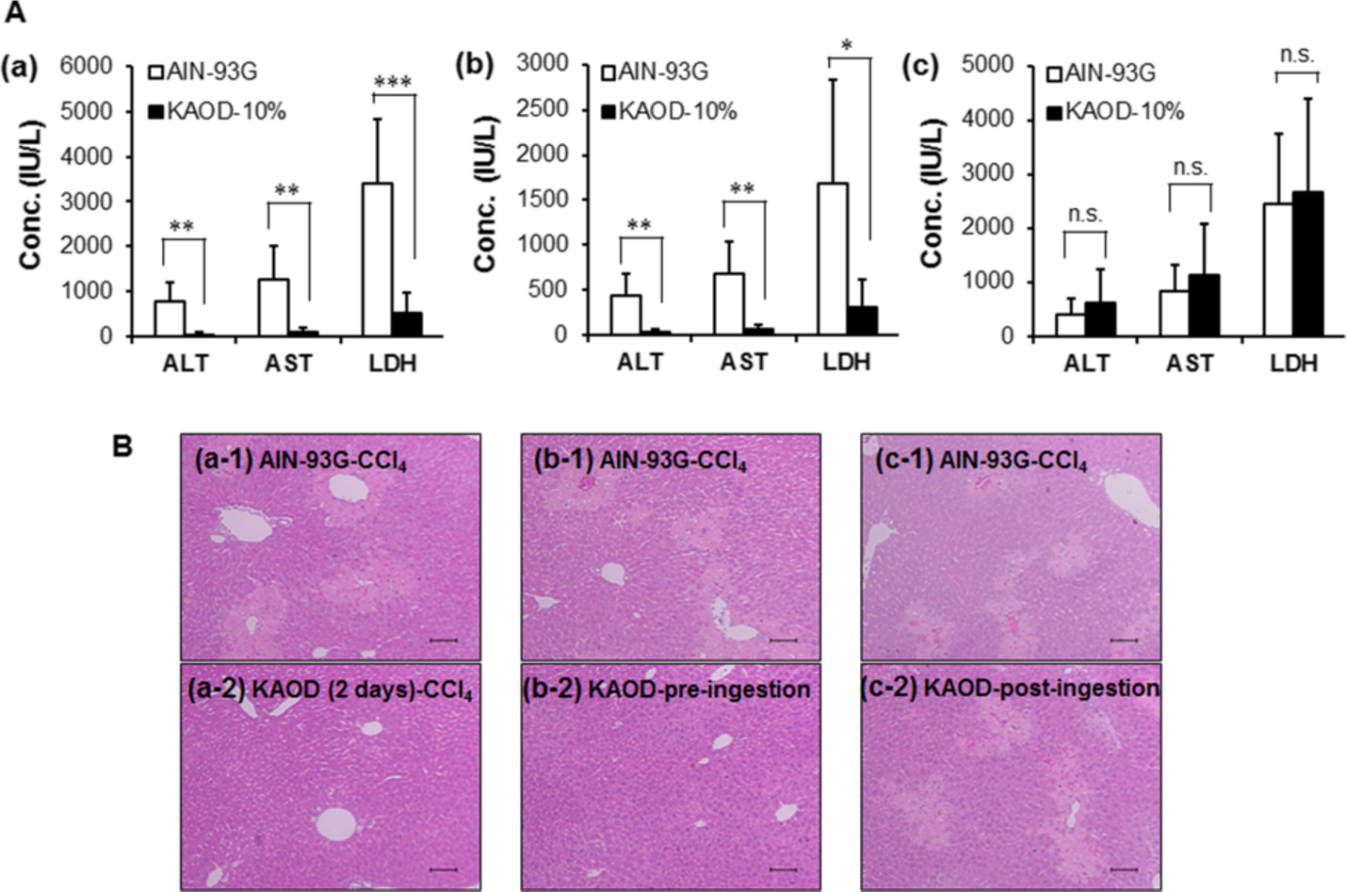
**Effective dosing period of KAOD on hepatoprotection.** Serum and livers were harvested from ICR mice fed KAOD-10% *ad libitum* from day 9 until the end of the experiment, from day 9 until CCl_4_ injection or from CCl_4_ injection until the end of the experiment. **(A)** The activities of ALT, AST, and LDH in the serum of KAOD-10%-administered mice (a) for 2 days, (b) for 24 h from day 9 until CCl_4_ injection or (c) for 24 h from CCl_4_ injection until the end of the experiment were measured using commercially available kits. Data are presented as mean ± standard deviation, *n* =6. Significant difference from AIN-93G: *p <0.05; **p <0.01; n.s.: not significant. **(B)** HE stained liver sections from mice administered (a-1) AIN-93G with CCl_4_ (control for 2 days), (a-2) KAOD-10% (2 days) with CCl_4_, (b-1) AIN-93G with CCl_4_ (control for pre-ingestion group), (b-2) KAOD-10% (pre-ingestion group) with CCl_4_, (c-1) AIN-93G with CCl_4_ (control for post-ingestion group), (c-2) KAOD-10% (post-ingestion group) with CCl_4_. Scale bar =100 μm.

To determine the optimal use of *A. brasiliensis*, we assessed the length of time taken for orally administered KAOD-10% to protect against CCl_4_-induced liver injury. Administration of KAOD-10% for 2 days was sufficient for hepatoprotective effects, assessed by the suppression of enzyme alterations (Figure 5A). Moreover, to assess whether the hepatoprotective effects of *A. brasiliensis* were preventive or curative, mice were treated with KAOD-10% for 24 h before or after injection with CCl_4_. Hepatoprotection was observed only in the pre-ingestion group. In addition, histopathological analyses strongly supported a hepatoprotective effect of KAOD-10% after 24 h of administration in the pre-ingestion group (Figure 5). These data suggest that the oral administration of KAOD exerts preventative effects against acute hepatic injury, and applies raid effects.

**Figure 6:**
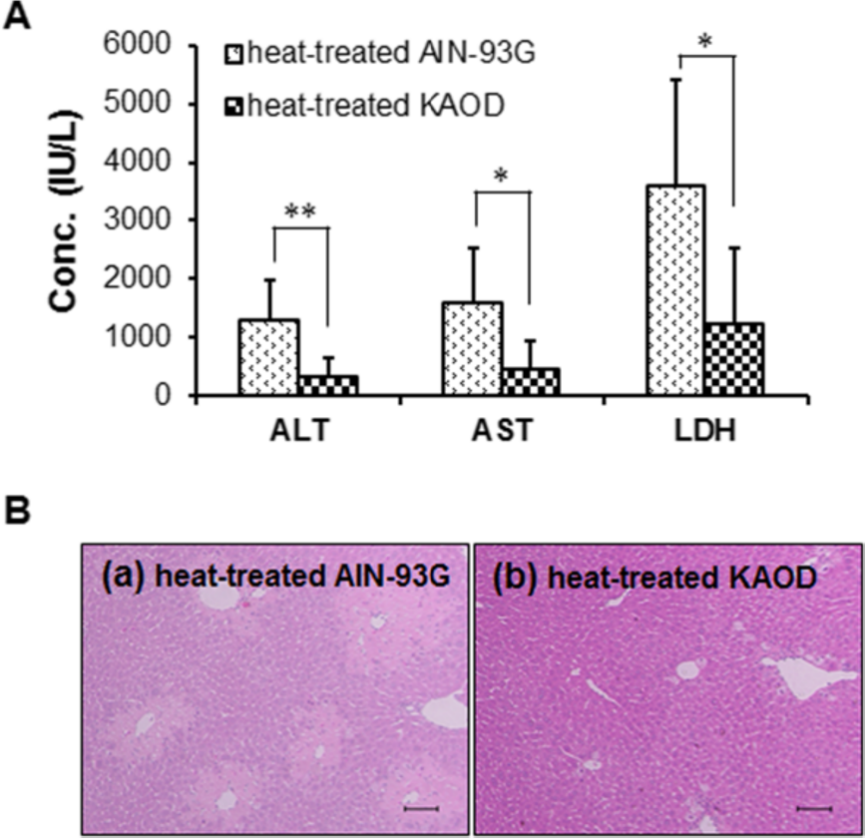
**Effect of heat treatment of KAOD on hepatoprotection.** Serum and livers were isolated from ICR mice fed heat-treated AIN-93G or heat-treated KAOD *ad libitum* from day 7. **(A)** The activities of ALT, AST, and LDH in serum were assessed using commercially available kits. Data are presented as mean ± standard deviation, *n* =6. Significant difference between heat-treated AIN-93G and heat-treated KAOD: *p <0.05; **p <0.01. **(B)** HE stained liver sections from mice administered (a) heat-treated AIN-93G with CCl_4_, and (b) heat-treated KAOD with CCl_4_ were observed under a normal light microscope. Scale bar =100 μm.

During the preparation of experimental diets containing KAOD, we avoided high temperatures to preserve a variety of heat-sensitive properties. Therefore, we next assessed the effects of heat processing on the hepatoprotective properties of *A. brasiliensis*. As shown in Figure 6, the hepatoprotective effects of orally administered KAOD-10% on the release of hepatic enzymes and the histopathological changes in the liver were not affected by autoclaving, suggesting that the hepatoprotective properties of *A. brasiliensis* are heat stable.

## Discussion

In 2008, a clinical study reported that *A. brasiliensis* extracts normalized liver function in hepatitis B patients [18]. In addition, several animal experiments have investigated the hepatoprotective effects of these mushroom extracts [19, 20]. However, the process of extraction not only concentrates the active ingredients, but also increases the potential for side effects. In 2006, possible adverse effects of *A. brasiliensis* were reported, where it was suggested that intake of these mushroom extracts may induce hepatic dysfunction [21]. Therefore, in this study we did not make extracts, and demonstrated the hepatoprotective activities of non-extracted fruiting bodies of *A. brasiliensis* using outdoor cultivation (Figures 3). In addition, because indoor-cultivated mushrooms failed to protect against liver damage (Figure 4), these mushrooms may require extraction or higher doses to exert hepatoprotective activities, which may increase the possibility of adverse effects. The safety of the normal use of non-extracted KAOD was demonstrated in our previous clinical study [6]. Therefore, outdoor cultivation could be better a method for reducing the risk of side effects. In addition, because hepatoprotective activities of KAOD were observed even when it was administered 24 h before CCl_4_ injection (Figure 5) and was not affected by autoclaving (Figure 6), the key molecules for hepatoprotection could be rapid-acting and heat-tolerant. Thus, these characteristics could be exploited to help use this mushroom for inhibiting hepatic damage.

Although indoor systematic cultivation has the advantage of being able to provide a stable supply, we believe that outdoor growth, and particularly sunshine, is one of the most important factors for the biological activity of this mushroom. Sunlight increased the levels of ingredients such as vitamin D and β-glucan, and enhanced the activity of several enzymes [15]. This study showed that although outdoor-cultivated *A. brasiliensis* contained more abundant β-glucan than that grown in the shade, the immunomodulatory effects of these mushrooms may be similar because the primary structure of β-glucan was unchanged. Nevertheless, many reports have suggested beneficial effects of vitamin D [22, 23]. In addition, other biological properties including enzymes and molecules produced by the enzymes could be affected by the cultivation conditions. Consistent with this, our study demonstrated that KAOD wields increased antioxidative activity compared with other mushrooms (Figure 2). Therefore, it is possible that outdoor cultivation could augment the antioxidative properties of *A. brasiliensis*, because the mushrooms protect themselves from sunlight-induced oxidative damage.

Many reports have described hepatoprotective properties of *A. brasiliensis*[24, 25], but the underlying mechanism(s) of these effects are yet to be elucidated. Because both JAID-10% and KAOD-1% failed to exert hepatoprotection, we considered if KAOD contained factors that would explain the 10-fold greater hepatoprotective properties than JAID. However, the basic ingredients of KAOD, KAID, and JAID were similar, except for vitamin D (Table 2), and we failed to demonstrate any contribution of vitamin D2 to hepatoprotection (data not shown). To clearly define the pharmacologic actions of *A. brasiliensis*, the molecular mechanism(s) behind the hepatoprotective effects of this mushroom must also be investigated.

Our current research focuses on the antioxidative components of *A. brasiliensis*, because the oral administration of several antioxidants protects against liver injury in animal models [26, 27]. Therefore, we will attempt to identify the key molecules that exert these hepatoprotective activities using both indoor- and outdoor-cultivated *A. brasiliensis* in future studies. The antioxidant activity of this mushroom is modulated not only by cultivation method, but also by different maturation phases [17]. Therefore, we propose that the pharmaceutical activity of *A. brasiliensis* could be optimized by the development of a basic manufacturing method.

## Conclusions

In the present study, we demonstrated that open-air cultivation strongly upregulated the total antioxidative activity of *A. brasiliensis*. In addition, outdoor-cultivated *A. brasiliensis* was more effective than mushrooms grown in the shade at protecting against CCl_4_-induced acute hepatic injury in mice. Therefore, we concluded that open-air cultivation could augment the antioxidative and hepatoprotective properties of the fruiting bodies of *A. brasiliensis*.

## Abbreviations

ALT: Alanine aminotransferase
AST: Aspartate aminotransferase
DPPH: 1,1-diphenyl-2-picrylhydrazyl
HE: Hematoxylin and eosin
LDH: Lactate dehydrogenase
TLR: Toll-like receptor.
